# Fatal Septic Shock Caused by Enterotoxigenic *Escherichia coli* O128 and Rare Polymicrobial Co-Infection with *Streptococcus equi* Subsp. *zooepidemicus*, *Klebsiella oxytoca* and *Enterococcus durans* in a Patient with Liver Cirrhosis: A Case Report

**DOI:** 10.3390/microorganisms14040750

**Published:** 2026-03-27

**Authors:** Petar Vasilev, Sema Chifchy, Aleksandar Ivanov, Vida Georgieva, Maria Radoslavova Pavlova, Yordan Kalchev, Mariyana Stoycheva

**Affiliations:** 1 Department of Infectious Diseases, Parasitology and Tropical Medicine, Faculty of Medicine, Medical University-Plovdiv, 4002 Plovdiv, Bulgaria; 2 Clinic for Infectious Diseases, St. George University Hospital, 4001 Plovdiv, Bulgaria; semanur-kesen@hotmail.com; 3 Department of General and Clinical Pathology, Medical University-Plovdiv, 4002 Plovdiv, Bulgaria; aleksandar.ivanov@mu-plovdiv.bg; 4 Department of Pathology and Cytopathology, St. George University Hospital, 4001 Plovdiv, Bulgaria; 5 Department of Medical Microbiology and Immunology “Prof. Dr. ElissayYanev”, Faculty of Pharmacy, Medical University-Plovdiv, 4002 Plovdiv, Bulgaria; vida.georgieva@mu-plovdiv.bg (V.G.); yordan.kalchev@mu-plovdiv.bg (Y.K.); 6 Laboratory of Microbiology, St. George University Hospital, 4002 Plovdiv, Bulgaria; 7 National Reference Laboratory of Enteric Infections, Pathogenic Cocci and Diphtheria, Department of Microbiology, National Centre of Infectious and Parasitic Diseases, 1504 Sofia, Bulgaria; mimipavlovaa@gmail.com; 8 Research Institute, Medical University-Plovdiv, 4002 Plovdiv, Bulgaria; marianavartigova49@gmail.com

**Keywords:** polymicrobial infection, septic shock, *Escherichia coli* O128, *Streptococcus equi* subsp. *zooepidemicus*, cirrhosis

## Abstract

*Escherichia coli*, *Streptococcus equi* subsp. *zooepidemicus*, *Klebsiella oxytoca*, and *Enterococcus durans* are microorganisms capable of causing severe infections, particularly in patients with underlying comorbidities or immune dysfunction. We report a rare clinical case of a 65-year-old man with advanced cardiac and hepatic disease who developed severe diarrheal syndrome followed by septic shock, rapid clinical deterioration, and death. Microbiological examination of autopsy specimens from the intestinal wall and spleen identified *Escherichia coli* O128 with an enterotoxigenic profile (*lt+*, *st+*, *eae−*), together with *Streptococcus equi* subsp. *zooepidemicus*, *Klebsiella oxytoca*, and *Enterococcus durans*. Histopathological analysis demonstrated catarrhal enteritis with fibrinous deposits, mucosal edema, vascular congestion, and inflammatory infiltration. Although the microbiological findings were partly derived from autopsy material and postmortem bacterial translocation cannot be completely excluded, the concordance between clinical presentation, laboratory findings, and morphological changes supports the presence of a clinically significant infectious process. To our knowledge, this is the first reported human case of fatal polymicrobial infection involving these four pathogens. The case highlights the potential severity of polymicrobial infections in patients with cirrhosis-associated immune dysfunction and underscores the importance of integrated microbiological and molecular diagnostics for accurate etiological assessment.

## 1. Introduction

According to the updated 2016 definitions, sepsis is defined as life-threatening organ dysfunction caused by a dysregulated host response to infection, whereas septic shock represents a subset of sepsis characterized by profound circulatory and metabolic abnormalities associated with increased mortality [1]. Sepsis and septic shock represent a continuum of progressive physiological instability in response to systemic infection [2].

Over recent decades, the incidence and severity of septic conditions have increased, largely due to the growing population of elderly patients, individuals with immune dysfunction, and patients with chronic comorbidities [3,4,5].

Polymicrobial infections represent a particularly challenging clinical entity. Interactions between different pathogens may amplify the inflammatory response and lead to more severe clinical courses compared with monomicrobial infections. Clinical studies indicate that polymicrobial sepsis is associated with higher rates of organ failure, prolonged hospitalization, and increased mortality [6]. Moreover, infections involving multiple pathogens have been identified as an independent prognostic factor for poor outcomes in severe gastrointestinal and intra-abdominal infections [6,7,8,9,10,11].

Zoonotic and zooanthroponotic infections may pose a significant threat in immunocompromised patients. In the presence of chronic conditions such as liver cirrhosis or heart failure, microorganisms primarily associated with animals may cause severe and life-threatening infections [12,13]. *Streptococcus equi* subsp. *zooepidemicus* is a well-recognized veterinary pathogen; although human infections are rare, they can present with severe manifestations including meningitis, endocarditis, sepsis, or septic shock [12,13].

*Escherichia coli* remains one of the most common bacterial causes of gastrointestinal infections. Pathogenic strains are classified into several pathotypes based on their virulence mechanisms, including enterotoxigenic (ETEC), enteropathogenic (EPEC), enteroinvasive (EIEC), enteroaggregative (EAEC), and Shiga toxin-producing (STEC/EHEC) variants [14,15]. Enterotoxigenic strains produce heat-labile (LT) and/or heat-stable (ST) enterotoxins that induce intestinal fluid secretion and watery diarrhea. In the present case, molecular analysis demonstrated an enterotoxigenic profile (*lt+*, *st+*, *eae*−), consistent with the ETEC pathotype and supporting a toxin-mediated mechanism of diarrheal disease.

Liver cirrhosis represents an independent risk factor for severe infection and poor outcome in sepsis. Cirrhosis-associated immune dysfunction increased intestinal permeability, and bacterial translocation significantly increased the risk of systemic infection and septic shock [16,17]. Despite advances in intensive care management, mortality among patients with cirrhosis and sepsis remains high [5,16,18,19].

In this context, we present a fatal case of polymicrobial infection involving *Escherichia coli* O128, *Streptococcus equi* subsp. *zooepidemicus*, *Klebsiella oxytoca*, and *Enterococcus durans* in a patient with advanced hepatic and cardiac disease. The aim of this report is to analyze the clinical course, microbiological and histopathological findings, and the diagnostic challenges associated with interpretation of microbiological results obtained partly from autopsy material.

To evaluate the novelty of this observation, a targeted literature search was performed in PubMed/MEDLINE, Scopus, and Google Scholar databases covering the period 2000–2024 using combinations of the following keywords: *Escherichia coli* O128, *Streptococcus equi* subsp. *zooepidemicus*, *Klebsiella oxytoca*, *Enterococcus durans*, polymicrobial infection, and sepsis. No previously published reports describing a fatal polymicrobial infection involving all four pathogens were identified.

## 2. Materials and Methods

### 2.1. Microbiological Investigation and Identification of Isolates

The autopsy was performed approximately 20 h after death. No blood cultures or additional microbiological specimens were obtained during life due to the rapid clinical deterioration and fatal outcome of the patient. Samples for microbiological analysis were collected immediately after opening the body cavities under standard aseptic conditions. Tissue specimens obtained during autopsy (intestinal wall and spleen) were subjected to routine culture-based microbiological investigation.

Samples were inoculated onto selective and enriched media, including 5% sheep blood agar, Levine eosin–methylene blue agar (EMB), and thioglycolate broth, and incubated aerobically at 35–37 °C for 24 h.

Primary identification of the isolated microorganisms was performed using MALDI-TOF mass spectrometry (Matrix-Assisted Laser Desorption/Ionization Time-of-Flight) with the VITEK^®^ MS system (bioMérieux, Marcy-l’Étoile, France). Additional phenotypic identification was carried out using the automated VITEK^®^ 2 system (bioMérieux, France). Results were interpreted according to current clinical microbiology standards, in accordance with EUCAST version 2024.

### 2.2. Molecular Confirmation of Escherichia coli O128

Molecular characterization of the *Escherichia coli* isolate was performed using real-time PCR with commercial diagnostic kits (CerTest BIOTEC, Zaragoza, Spain), according to the manufacturer’s instructions. The analysis targeted genes encoding the heat-labile enterotoxin (*lt*) and heat-stable enterotoxin (*st1*).

The presence of virulence genes was confirmed using the internal positive control included in the kit, as well as an external positive control consisting of a reference enterotoxigenic *E. coli* (ETEC) strain provided through an external quality assessment (EQA) program by Statens Serum Institut (SSI), Copenhagen S, Denmark.

Phenotypic serological confirmation of the serotype was performed by slide agglutination using anti-*E. coli* antisera (Sifin, Berlin, Germany). Initially, polyvalent reagents (Anti-Coli I, II, and III) were applied, followed by the monospecific reagent Anti-Coli O128:K67 for definitive serotype identification.

### 2.3. Antimicrobial Susceptibility Testing

Antimicrobial susceptibility testing was performed using the automated VITEK^®^ 2 Compact system (bioMérieux, France). Results were interpreted according to the European Committee on Antimicrobial Susceptibility Testing (EUCAST, Växjö, Sweden) 2024 breakpoints. Susceptibility was categorized as susceptible (S), susceptible with increased exposure (I), or resistant (R), in accordance with current EUCAST standards.

### 2.4. Interpretation of Microbiological Findings and Postmortem Considerations

During interpretation of microbiological results, the postmortem interval between death and sample collection was taken into account, along with the potential impact of postmortem microbiological changes. Specimens from the intestinal wall and spleen were collected immediately after opening of the body cavities under sterile conditions, using sterile instruments and without delay in processing, following routine microbiological protocols.

The time of sampling and working conditions were documented to support subsequent interpretation of results in the context of possible postmortem processes.

Particular attention was paid to distinguishing clinically significant infection from possible postmortem contamination by correlating microbiological findings with clinical presentation, laboratory data obtained during life, and histopathological evidence of inflammatory intestinal lesions.

### 2.5. Ethical Considerations

The autopsy was performed for medical indications in accordance with applicable national legislation and the internal regulations of St. George University Hospital, Plovdiv. According to institutional policies and national regulations, additional ethics committee approval was not required, as the study represents a retrospective analysis of clinical and laboratory data obtained within routine diagnostic and therapeutic practice, including autopsy performed for medical reasons.

The patient had signed informed consent upon hospital admission for diagnostic and therapeutic procedures in accordance with standard hospital practice. All data used in this study were anonymized, and confidentiality of personal information was strictly maintained.

## 3. Case Presentation

A 65-year-old male patient (Y.I.P., Medical Record No. 26392/2024) was admitted to the Clinic of Infectious Diseases, St. George University Hospital, Plovdiv, with a diagnosis of acute intestinal infection. The chronological sequence of clinical events from symptom onset to death is summarized in Table 1. He presented with an intense diarrheal syndrome of 1–2 days’ duration (≥10 watery, light-brown stools daily without mucus or blood), generalized weakness, chest and abdominal pain, and fever. One day prior to symptom onset, he had consumed green salad and lamb meat.

His medical history was significant for chronic heart failure associated with arterial hypertension and previous placement of two coronary stents; liver cirrhosis with complications (portal hypertension and esophageal varices); and anemia.

Upon admission, the patient was in moderately impaired general condition, afebrile, conscious, and oriented, but psychomotor-agitated. Clinical findings were consistent with toxic-infectious and diarrheal syndrome. He was moderately intoxicated and dehydrated (grade I–II), with pale, cold, and sweaty skin of reduced turgor. Petechiae, ecchymoses, and suffusions were observed on the upper extremities. The tongue was dry and coated with a white layer. Arthralgia and myalgia were present.

Respiratory rate was 40 breaths/min, with oxygen saturation of 94% in room air and vesicular breath sounds without pathological findings. Heart rate was 98 beats/min with arrhythmic cardiac activity; arterial blood pressure was 114/75 mmHg. The abdomen was distended above chest level, diffusely tender but allowing deep palpation. Increased bowel peristalsis and bilateral lower limb edema were noted. Neurological examination was unremarkable.

Intravenous rehydration therapy was initiated immediately. Despite treatment, the patient’s condition deteriorated rapidly. He developed coma (Glasgow Coma Scale score: 3), progressive tachypnea (40–45 breaths/min), marked respiratory distress, and peripheral lividity. Advanced cardiopulmonary resuscitation was performed in full scope, in collaboration with an intensivist; however, resuscitative efforts were unsuccessful, and death ensued.

### 3.1. Microbiological Investigations Performed During Life

At admission, stool culture was obtained within the first hour of hospitalization, approximately two hours before death. Microbiological analysis did not demonstrate growth of pathogenic enteric microorganisms. The negative result may be explained by the very early sampling relative to symptom onset, the short interval between hospitalization and death, and the possibility that the causative pathogens were present predominantly in the intestinal mucosa rather than in the stool at the time of sampling. Due to the rapid clinical deterioration and the short interval to death (approximately 3 h), blood cultures were not obtained.

### 3.2. Laboratory Findings

Hematological parameters (Table 2) revealed mild anemia (hemoglobin 129 g/L; red blood cells 3.67 × 10^12^/L) and thrombocytopenia (platelets 90 × 10^9^/L), likely associated with hemorrhagic diathesis and/or consumptive coagulopathy. The erythrocyte sedimentation rate (ESR) was elevated (33 mm/h). Differential leukocyte count showed neutrophilia (87%) with relative lymphopenia (9%) and monocytopenia.

### 3.3. Coagulation Parameters

Hemostatic assessment (Table 3) demonstrated markedly reduced prothrombin time activity (PT 27.2%) and a markedly elevated D-dimer level (>35 mg/L), suggesting pronounced activation of coagulation, consistent with thrombotic risk and/or disseminated intravascular coagulation (DIC).

### 3.4. Biochemical Findings

Biochemical analysis (Table 4) revealed hyperbilirubinemia (total bilirubin 56.7 µmol/L; direct bilirubin 18.7 µmol/L), markedly reduced serum cholinesterase activity (2000 U/L), indicating impaired hepatic synthetic function, and markedly elevated AST (542 U/L). Inflammatory activity was reflected by elevated C-reactive protein (114 mg/L). Increased CK-MB (478 U/L) and troponin I (0.08 ng/mL) suggested acute myocardial ischemia in the context of underlying cardiac disease and a systemic inflammatory response. Renal parameters were also impaired (creatinine 163 µmol/L; urea 11.6 mmol/L).

### 3.5. Clinical Course and Management

Due to the extremely rapid clinical deterioration and death occurring shortly after admission, no antimicrobial therapy was initiated. The patient developed fulminant hemodynamic instability and coma, which precluded targeted treatment. Inotropic support was not initiated because circulatory collapse progressed rapidly before stabilization could be achieved. Cardiopulmonary resuscitation was performed according to standard advanced life support protocols.

### 3.6. Postmortem Microbiological Findings

**Figure 1:**
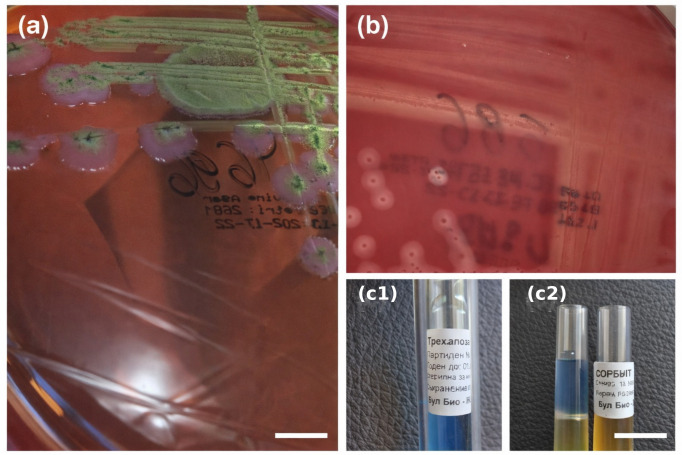
Microbiological culture findings from intestinal autopsy specimens. (**a**) Growth of *Escherichia coli* on LevineEMB agar; (**b**) colonies of *Streptococcus equi* subsp. *zooepidemicus* isolated from intestinal tissue on blood agar; (**c1**,**c2**) biochemical testing used for species differentiation, including thioglycolate broth and sorbitol fermentation test. The blue coloration (**c1**) indicates a negative reaction, while yellow coloration (**c2**) indicates a positive fermentation reaction. The non-English text visible on the tubes corresponds to the original labeling of the reagents (trehalose and sorbitol).

From intestinal autopsy specimens, *Escherichia coli* (Figure 1a) and *Streptococcus equi* subsp. *zooepidemicus* (Figure 1b,c1,c2) were isolated. The *E. coli* isolate was susceptible to ampicillin, piperacillin, cefoxitin, cefotaxime, ceftriaxone, amoxicillin-clavulanic acid, amikacin, ciprofloxacin, trimethoprim/sulfamethoxazole, levofloxacin, meropenem, imipenem, cefepime, and tigecycline (Table 5). The *S. equi* subsp. *zooepidemicus* isolate was susceptible to penicillin, ceftriaxone, cefepime, vancomycin, teicoplanin, trimethoprim/sulfamethoxazole, tigecycline, linezolid, moxifloxacin, amoxicillin-clavulanic acid, cefuroxime (axetil), and rifampin; resistant to erythromycin, clindamycin, and tetracycline; and categorized as susceptible with increased exposure to levofloxacin (Table 6).

From spleen tissue, *Enterococcus durans* and *Klebsiella oxytoca* were isolated. *E. durans* was susceptible to ampicillin, ciprofloxacin, norfloxacin, gentamicin, vancomycin, teicoplanin, linezolid, tigecycline, amoxicillin-clavulanic acid, piperacillin, levofloxacin, and ampicillin-sulbactam (Table 7). *K. oxytoca* was susceptible to cefoxitin, cefotaxime, ceftriaxone, amoxicillin-clavulanic acid, amikacin, ciprofloxacin, trimethoprim/sulfamethoxazole, levofloxacin, meropenem, imipenem, cefepime, piperacillin-tazobactam, colistin, and ceftazidime/avibactam, and resistant to ampicillin and piperacillin (Table 8).

### 3.7. Molecular Findings

The intestinal isolate was confirmed as enterotoxigenic *E. coli* O128 harboring *lt* and *st* toxin genes, without the adhesion gene *eae*.

### 3.8. Autopsy and Histopathology

Autopsy revealed fibrinous deposits on the serosa of the small intestine, mucosal edema, and advanced atherosclerotic changes in the aorta (stage III), consistent with chronic ischemic heart disease and pulmonary edema. The principal morphological findings contributing to death included micronodular liver cirrhosis with portal hypertension, esophageal varices, and gastric hemorrhage.

**Figure 2:**
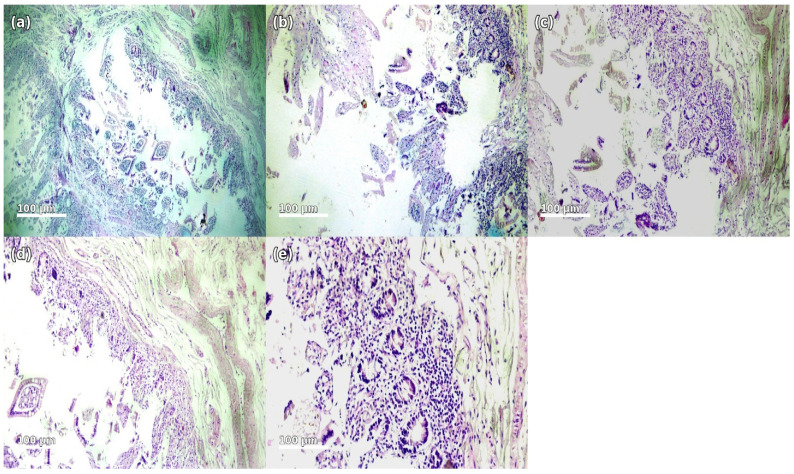
Histopathological findings of the small intestine (H&E staining, magnification ×100–400): (**a**) Section of the small intestinal wall demonstrating edema and focal inflammatory infiltration. (**b**) Catarrhal enteritis characterized by prominent mucosal swelling, fibrinous exudate, and dense infiltration by polymorphonuclear leukocytes. (**c**) Fibrinous plaques and degenerative epithelial changes with accumulation of inflammatory cells. (**d**) Pronounced inflammatory infiltration of the lamina propria with areas of epithelial desquamation. (**e**) Edematous intestinal wall with vascular congestion and diffuse inflammatory response. The images are presented using standard hematoxylin and eosin (H&E) staining, where hematoxylin stains cell nuclei blue-purple and eosin stains cytoplasm and extracellular components in varying shades of pink. These color characteristics are inherent to the staining method and facilitate the visualization of tissue architecture and inflammatory changes.

Histological examination of the small intestine showed acute catarrhal enteritis with epithelial desquamation and superficial degenerative changes covered by fibrinoleukocytic exudate forming fibrinous plaques. The lamina propria was markedly edematous with dense inflammatory infiltrate composed predominantly of polymorphonuclear leukocytes, lymphocytes, and scattered macrophages. Vascular congestion with dilated capillaries and venules and interstitial edema were observed. Focal superficial epithelial necrosis was present without transmural involvement. These findings are consistent with an acute exudative infectious–inflammatory process. The histopathological features support the presence of an acute infectious–inflammatory process affecting the intestinal mucosa, consistent with the clinical presentation of severe diarrheal disease. Representative histological features are shown in Figure 2.

## 4. Discussion

The present clinical case describes a rare polymicrobial infection with a fulminant course and fatal outcome in a 65-year-old patient with advanced liver cirrhosis and significant cardiac pathology. The disease was characterized by rapid progression from acute intestinal infection with severe diarrheal syndrome to septic shock and multiorgan dysfunction. The isolation of four microorganisms—enterotoxigenic *Escherichia coli* O128, *Streptococcus equi* subsp. *zooepidemicus*, *Klebsiella oxytoca*, and *Enterococcus durans*—suggests a complex infectious process with potential synergistic interaction between pathogens. In this polymicrobial context, enterotoxigenic *E. coli* O128 was likely the primary driver of the acute diarrheal syndrome, while the remaining microorganisms may have contributed to systemic dissemination and amplification of the inflammatory response. To our knowledge, this is the first reported fatal polymicrobial infection involving this specific combination of pathogens in a human patient.

Polymicrobial infections are associated with a higher risk of septic shock, multiorgan failure, and mortality compared with monomicrobial infections [20,21,,22]. The number of involved microorganisms has been considered an independent prognostic factor for adverse outcomes [21,22]. In the present case, the combination of four pathogens likely contributed to an amplified systemic inflammatory response and rapid hemodynamic decompensation.

The enterotoxigenic profile of the isolated *E. coli* O128 (*lt*+, *st*+, *eae*−) provides a pathophysiological explanation for the severe diarrheal syndrome through toxin-mediated hypersecretion of water and electrolytes. Dehydration, in combination with systemic inflammatory activation, likely accelerated progression to septic shock. The negative stool culture obtained during life does not exclude an infectious etiology, as enterotoxigenic strains induce diarrhea primarily via toxin-mediated mechanisms without obligatory mucosal invasion.

The simultaneous isolation of four microorganisms from intestinal and splenic autopsy specimens should be interpreted cautiously. In the absence of ante-mortem blood cultures or molecular evidence of hematogenous dissemination, definitive proof of systemic polymicrobial bloodstream infection cannot be established. Nevertheless, the overall pattern of findings suggests that the microorganisms were not merely incidental contaminants. Enterotoxigenic *Escherichia coli* O128, confirmed by virulence gene detection (*lt*+, *st*+, *eae*−), was most likely the primary driver of the acute secretory diarrheal syndrome and the initial trigger of systemic deterioration. In contrast, *Streptococcus equi* subsp. *zooepidemicus*, *Klebsiella oxytoca*, and *Enterococcus durans* may have acted as secondary opportunistic contributors in the setting of cirrhosis-associated immune dysfunction, increased intestinal permeability, and possible bacterial translocation. Thus, while the precise sequence of dissemination cannot be proven, the microbiological findings are most consistent with a clinically significant polymicrobial infectious process rather than isolated postmortem contamination alone.

Human infections caused by *Streptococcus equi* subsp. *zooepidemicus* are rare but often severe, frequently presenting with sepsis and systemic complications [16]. *Klebsiella* spp., including *K. oxytoca*, possess zoonotic potential and have been identified in livestock and animal-derived food products, although the extent of dissemination in animal husbandry and the mechanisms of interspecies transmission remain incompletely defined [23]. Although the patient reported consumption of lamb meat prior to symptom onset, the exact source of infection cannot be determined and foodborne exposure should therefore be considered only a hypothetical possibility. *Enterococcus durans* is primarily opportunistic; its isolation from splenic tissue supports the systemic nature of the infection.

Liver cirrhosis represents a key aggravating factor. Patients with cirrhosis develop cirrhosis-associated immune dysfunction, characterized by impaired intestinal barrier function, increased intestinal permeability, and a predisposition to bacterial translocation [18,19]. Cirrhosis is associated with increased incidence of sepsis and multiorgan failure, particularly in the presence of a gastrointestinal infectious focus [24,25]. These mechanisms likely facilitated systemic dissemination and contributed to the unfavorable outcome.

Morphological findings of acute inflammatory changes in the intestinal wall, including fibrinous exudate and neutrophilic infiltration, support an infectious etiology. Although chronic alterations consistent with cirrhosis and portal hypertension were present, the histopathological pattern corresponded to an active inflammatory process occurring during life. The clinical course—rapid progression from gastrointestinal symptoms to hemodynamic instability—together with laboratory evidence of coagulation abnormalities and markedly elevated D-dimer levels, is consistent with septic shock complicated by disseminated intravascular coagulation rather than primary hepatic decompensation. Primary hepatic decompensation alone appears less likely to explain the fulminant presentation, because the dominant initial manifestations were acute watery diarrhea, toxic-infectious syndrome, rapid hemodynamic collapse, and laboratory evidence of systemic inflammatory and coagulation activation. At the same time, advanced cirrhosis undoubtedly constituted a major predisposing and aggravating factor that lowered the threshold for rapid progression to multiorgan failure.

The absence of transmural invasion does not exclude a fulminant clinical course. In the present case, severe toxin-mediated secretory diarrhea caused by enterotoxigenic *E. coli* likely resulted in rapid fluid loss, hemodynamic instability, and systemic inflammatory activation.

### 4.1. Interpretation of Microbiological Findings in the Context of Postmortem Translocation

Interpretation of autopsy microbiological results requires caution due to the possibility of postmortem bacterial translocation. After cessation of circulation, the barrier function of the intestinal wall progressively deteriorates, potentially allowing passive dissemination of microorganisms to internal organs. Nevertheless, the presence of inflammatory histopathological changes in the intestinal mucosa, together with clinical evidence of systemic inflammatory response prior to death, argues against a purely postmortem origin of the detected microorganisms.

In the present case, however, the isolation of microorganisms from the spleen—an organ expected to be sterile during life—must be interpreted in conjunction with the full clinicopathological context. The splenic findings alone cannot prove ante-mortem hematogenous dissemination, and selective postmortem contamination cannot be completely excluded. Nevertheless, the combination of (i) severe diarrheal syndrome with fulminant deterioration during life, (ii) marked inflammatory and coagulation abnormalities, (iii) histopathological evidence of acute intestinal infectious–inflammatory changes, and (iv) demonstration of enterotoxigenic virulence determinants in *E. coli* O128 supports the interpretation that infection played a clinically significant role in the terminal event. Therefore, the splenic isolates are interpreted cautiously as possible indicators of systemic spread or advanced bacterial translocation in a highly vulnerable patient, rather than definitive proof of bloodstream dissemination.

### 4.2. Clinical Implications

This case underscores the importance of an integrated diagnostic approach in high-risk patients, particularly those with advanced liver cirrhosis and acute diarrheal syndrome. In such patients, early clinical recognition of rapid dehydration, systemic inflammatory deterioration, and coagulation abnormalities is critical. From a laboratory perspective, prompt collection of ante-mortem microbiological specimens, including blood cultures and stool samples before clinical collapse whenever feasible, is essential for more reliable etiological interpretation. Molecular detection of virulence determinants may provide important complementary information when conventional cultures are negative or inconclusive. Clinically, the case also supports careful dietary assessment and counseling in cirrhotic patients, especially with regard to potentially contaminated animal-derived foods, as well as a low threshold for early supportive and empiric antimicrobial management in rapidly progressive suspected enteric infections.

### 4.3. Limitations

This clinical case has several limitations. The primary limitation is the absence of blood cultures obtained during life due to rapid clinical deterioration and the short interval before death, which complicates definitive differentiation between true systemic infection and possible postmortem bacterial translocation. The negative stool culture at admission may be explained by the toxin-mediated mechanism of enterotoxigenic *E. coli*, in which the bacterial load in stool may be low or transient. Autopsy was performed approximately 20 h after death, introducing a theoretical possibility of postmortem microbiological changes. The presence of severe comorbidities, including advanced liver cirrhosis and cardiac disease, represents an additional confounding factor in interpretation of the causal relationships. As a single case report, this study is inherently limited in terms of generalizability.

## 5. Conclusions

This case report describes a fatal polymicrobial infection involving *Escherichia coli* O128, *Streptococcus equi* subsp. *zooepidemicus*, *Klebsiella oxytoca*, and *Enterococcus durans* in a patient with advanced liver cirrhosis and severe comorbidities. The combination of enterotoxigenic diarrheal disease pathogens and cirrhosis-associated immune dysfunction and possible secondary polymicrobial translocation or systemic spread likely contributed to the rapid progression to septic shock and death.

The case highlights the value of an integrated diagnostic approach combining clinical evaluation, microbiological investigation, molecular characterization, and histopathological assessment. Although the interpretation is limited by the absence of ante-mortem blood cultures and by the postmortem interval before autopsy, the overall concordance of clinical, laboratory, microbiological, and morphological findings supports infection as the most plausible principal contributor to the fatal outcome.

## Figures and Tables

**Table 1:** Timeline of clinical events from symptom onset to death.

Time Point	Clinical Events
Day −2 to −1	Onset of intense watery diarrhea (≥10 episodes daily), weakness, abdominal and chest pain
Day −1	Consumption of green salad and lamb meat
Day 0—admission	Admission to the Clinic of Infectious Diseases with severe diarrheal syndrome and dehydration
First hour after admission	Stool culture obtained
~3 h after admission	Rapid clinical deterioration with respiratory distress and coma
Same day	Cardiopulmonary resuscitation performed; death occurred
~20 h postmortem	Autopsy and microbiological sampling

**Table 2:** Hematological parameters at admission (14 April 2024).

Parameters ^1^	14.04.2024	Reference Values
HGB	129	140–180 g/L
RBC	3.67	4.5–6 × 10^12^/L
HCT	0.403	0.40–0.54
WBC	3.81	3.5–10.5 × 10^9^/L
PLT	90	140–400 × 10^9^/L
ESR	33	2–25 mm/h
Neu	87	42–70%
Ly	9	22–48%
Mo	2	6–12%
Eo	2	0–6%
Ba	0	0–2%

^1^ Hemoglobin—HGB; Erythrocytes—RBC; Hematocrit—HCT; Leukocytes—WBC; Platelets—PLT; Erythrocyte Sedimentation Rate—ESR; Neutrophils—Neu; Lymphocytes—Ly; Monocytes—Mo; Eosinophils—Eo; Basophils—Ba.

**Table 3:** Hemostasis at admission (14 April 2024).

Parameters ^1^	14 April 2024	Reference Values
PT, %	27.2	70–120%
INR	1.88	
PT, sec	20.2	
APTT	34.5	24–35 s
TT	19	14–21 s
D-DIM	>35	0–0.50 mg/L
FBC	3.57	2.0–4.5 g/L

^1^ Prothrombin Time—PT; International Normalized Ratio—INR; Activated Partial Thromboplastin Time—APTT; Thrombin Time—TT; D-dimer—D-DIM; Fibrinogen—FBC.

**Table 4:** Biochemistry at admission (14 April 2024).

Parameters ^1^	14 April 2024	Reference Values
T BIL	56.7	3.4–21 μmol/L
D BIL	18.7	0.8–8.5 μmol/L
CHE	2000	3600–11,500 U/L
ALP	81	40–120 U/L
GGT	50	0–55 U/L
ALT	75	0–50 U/L
AST	542	0–50 U/L
Na	133	136–151 mmol/L
K	4.3	3.5–5.6 mmol/L
Cl	98	96–110 mmol/L
T PROT	77.7	60–83 g/L
ALB	31	35–52 g/L
CRP	114	0–10 mg/L
TnI	0.08	0.0–0.04 ng/mL
CK-MB	478	0–22 U/L
GLUC	6.6	2.8–6.1 mmol/L
CREA	163	74–134 μmol/L
UREA	11.6	3.2–8.2 mmol/L

^1^ Total Bilirubin—T BIL; Direct Bilirubin—D BIL; Serum Cholinesterase—CHE; Alkaline Phosphatase—ALP; Gamma-Glutamyl Transferase—GGT; Alanine Aminotransferase—ALT; Aspartate Aminotransferase—AST; Sodium—Na; Potassium—K; Chloride—Cl; Total Protein—T PROT; Albumin—ALB; C-Reactive Protein—CRP; Troponin—TnI; Creatine Kinase MB Fraction—CK-MB; Glucose—GLUC; Creatinine—CREA; Urea—UREA.

**Table 5:** Antimicrobial susceptibility profile of *Escherichia coli* isolated from intestinal tissue (Vitek-2 Compact, bioMérieux, France).

-	*E. coli*	Interpretation
1	Ampicillin	S *
2	Piperacillin	S
3	Cefoxitin	S
4	Cefotaxime	S
5	Ceftriaxone	S
6	Cefepime	S
7	Amoxicillin-clavulanic acid	S
8	Amikacin	S
9	Ciprofloxacin	S
10	Trimethoprim/sulfamethoxazole	S
11	Levofloxacin	S
12	Meropenem	S
13	Imipenem	S
14	Tigecycline	S

* S—susceptible.

**Table 6:** Antimicrobial susceptibility profile of *Streptococcus equi* subsp. *zooepidemicus* isolated from intestinal tissue (Vitek-2 Compact, bioMérieux, France).

-	*Streptococcus zooepidemicus*	Interpretation
1	Ceftriaxone	S *
2	Cefepime	S
3	Amoxicillin-clavulanic acid	S
4	Penicillin	S
5	Erythromycin	R **
6	Linezolid	S
7	Cefuroxime (axetil)	S
8	Trimethoprim/sulfamethoxazole	S
9	Levofloxacin	I ***
10	Vancomycin	S
11	Teicoplanin	S
12	Tetracycline	R
13	Clindamycin	R
14	Moxifloxacin	S
15	Tigecycline	S
16	Rifampin	S

* S—susceptible; ** I—intermediate susceptibility; *** R—resistant.

**Table 7:** Antimicrobial susceptibility profile of *Enterococcus durans* isolated from splenic tissue (Vitek-2 Compact, bioMérieux, France).

-	*Enterococcus durans*	Interpretation
1	Ampicillin	S *
2	Piperacillin	S
3	Amoxicillin-clavulanic acid	S
4	Norfloxacin	S
5	Linezolid	S
6	Ciprofloxacin	S
7	Levofloxacin	S
8	Vancomycin	S
9	Teicoplanin	S
10	Gentamycin	S
11	Ampicillin-sulbactam	S
12	Tigecycline	S

* S—susceptible.

**Table 8:** Antimicrobial susceptibility profile of *Klebsiella oxytoca* isolated from splenic tissue (Vitek-2 Compact, bioMérieux, France).

-	*Klebsiella oxytoca*	Interpretation
1	Ampicillin	R **
2	Piperacillin	R
3	Cefoxitin	S *
4	Cefotaxime	S
5	Ceftriaxone	S
6	Cefepime	S
7	Amoxicillin-clavulanic acid	S
8	Colistin	S
9	Ciprofloxacin	S
10	Trimethoprim/sulfamethoxazole	S
11	Levofloxacin	S
12	Meropenem	S
13	Imipenem	S
14	Amikacin	S
15	Ceftazidime/avibactam	S
16	Piperacillin-tazobactam	S

* S—susceptible; ** R—resistant.

## Data Availability

The data supporting the findings of this study are available from the corresponding author upon reasonable request.
